# Exploring the multifunctionality of myco-synthesized selenium nanoparticles: biological, docking study and plant growth-promoting perspectives

**DOI:** 10.3389/fmicb.2025.1565907

**Published:** 2025-03-14

**Authors:** Hana Sonbol, Eman Zakaria Ahmed, Eslam T. Mohamed, Asmaa F. Abdelmonem, Heba El-Sayed

**Affiliations:** ^1^Department of Biology, College of Science, Princess Nourah Bint Abdulrahman University, Riyadh, Saudi Arabia; ^2^Botany and Microbiology Department, Faculty of Science, Helwan University, Helwan, Egypt

**Keywords:** selenium nanoparticles, mycosynthesis, *Pleurotus ostreatus*, antioxidant, antimicrobial, antiviral, *Triticum aestivum*, molecular docking

## Abstract

Selenium is a vital trace mineral that regulates essential physiological functions, and the development of sustainable methods for synthesizing selenium nanoparticles (SeNPs) is an active area of research. This study reported the mycosynthesis of SeNPs using the aqueous extract of the edible mushroom *Pleurotus ostreatus*. The synthesized SeNPs were characterized using various analytical techniques, including UV–visible spectroscopy, dynamic light scattering, transmission electron microscopy (TEM), and Fourier-transform infrared spectroscopy (FTIR). The results revealed that the SeNPs exhibited spherical morphology with a diameter range of 72–148 nm, moderate stability with a zeta potential of −10.5 mV, and a polydispersity index of 0.27. The myco-synthesized SeNPs demonstrated potent antioxidant activity with a DPPH radical scavenging IC_50_ value of 662.1 ± 1.05 μg/mL, comparable to the standard antioxidant Trolox (IC_50_ = 24.42 ± 0.87 μM). Furthermore, SeNPs exhibited considerable antimicrobial efficacy against *Staphylococcus aureus* (17 ± 0.02 mm inhibition zone), followed by *Escherichia coli* (16 ± 1.04 mm) and *Candida albicans* (12 ± 0.3 mm). Additionally, SeNPs displayed moderate antiviral activity against a low-pathogenic coronavirus (229E) strain, with a selectivity index (SI) of 5. In agriculture, the application of SeNPs at 10 μM significantly increased primary metabolite production in wheat (*Triticum aestivum*), with total soluble sugars reaching 54.32 mg/g and soluble proteins increasing to 139.66 mg/g, promoting both shoot and root growth. The comprehensive characterization and evaluation of SeNPs provide valuable insights into their multifunctionality, paving the way for further exploration in medicine, agriculture, and environmental applications.

## 1 Introduction

Nanotechnology has emerged as a transformative field with diverse applications across multiple scientific and industrial domains (Bhardwaj et al., 2020). In medicine, nanoparticles are widely utilized for targeted drug delivery, imaging, and antimicrobial treatments, improving therapeutic efficacy and patient outcomes (Canaparo et al., 2019). In agriculture, nanomaterials enhance plant growth, increase nutrient uptake, and serve as effective agents for pest control, contributing to sustainable farming practices (Singh et al., 2021). In environmental science, nanotechnology plays a crucial role in water purification, pollutant degradation, and air filtration, mitigating environmental contamination (Naskar et al., 2022). Additionally, in industrial applications, nanomaterials enhance the mechanical properties of materials, improve energy storage systems, and contribute to the development of lightweight, durable composites (Hassan et al., 2021). These wide-ranging applications underscore the significance of nanotechnology in advancing scientific innovation and addressing global challenges.

The excessive utilization of synthetic chemicals, including antimicrobials, antivirals, and plant fertilizers, has raised significant concerns due to their environmental and health impacts (Abdelsalam et al., 2023). The indiscriminate use of antimicrobial agents has contributed to the emergence of resistant pathogens, disrupting microbial ecosystems and posing a substantial threat to public health. Meanwhile, excessive application of synthetic fertilizers and pesticides has been associated with soil degradation, water contamination, and ecological imbalances. Thus, there is an urgent need for sustainable and environmentally friendly alternatives that minimize these risks while maintaining efficacy in both pathogen control and agricultural productivity (Bahrulolum et al., 2021).

Selenium nanoparticles (SeNPs) offer a promising solution by acting as both antimicrobial agents and plant growth enhancers (Saravanan et al., 2021). SeNPs have been shown to exhibit significant antimicrobial activity against clinically relevant pathogens such as *S. aureus*, *E. coli*, and *C. albicans*. By reducing the reliance on conventional antimicrobials, SeNPs help lower the risk of antimicrobial resistance while minimizing pharmaceutical waste and environmental contamination (Nowruzi et al., 2023). Their controlled antimicrobial action makes them an effective alternative to traditional chemical treatments, ensuring a more sustainable approach to pathogen control (Abdelsalam et al., 2023).

In addition to their role in pathogen mitigation, SeNPs also function as bio-stimulants that enhance plant growth, nutrient uptake, and stress tolerance. Unlike conventional fertilizers that often contribute to nutrient leaching and environmental pollution, SeNPs offer a controlled-release mechanism that minimizes excessive chemical input while maximizing plant benefits. The integration of SeNPs into agricultural practices holds great potential for sustainable crop production by promoting plant health without the harmful consequences associated with synthetic fertilizers (Bahrulolum et al., 2021).

Selenium is a vital trace mineral that is integral to numerous physiological processes in the human body. It participates in the control of antioxidant defense mechanisms, thyroid function, and immunological response (Fernández-Lázaro et al., 2020). In the last few years, the production of selenium nanoparticles (SeNPs) has attracted significant interest owing to their enhanced bioavailability, improved therapeutic efficacy, and reduced toxicity compared to bulk selenium (Bisht et al., 2022).

The discovery and implementation of sustainable and environmentally friendly techniques for the production of SeNPs is a focus of ongoing study. One promising approach is the use of fungal extracts, a process known as “mycosynthesis,” which harnesses the metabolic capabilities of fungi to produce SeNPs (Xu, 2020). Fungi are known to secrete a variety of extracellular enzymes, proteins, and other biomolecules that can reduce and stabilize selenium ions, leading to the formation of SeNPs (Nayak et al., 2021).

The mycosynthesis of SeNPs offers several advantages, including the use of renewable and eco-friendly resources, mild reaction conditions, and the potential for scalable production (Zambonino et al., 2021). Furthermore, the fungal-derived SeNPs may exhibit unique physicochemical properties, such as enhanced stability, improved biocompatibility, and targeted delivery, which can be exploited for various applications in the fields of medicine, agriculture, and environmental remediation (Loshchinina et al., 2022).

The oyster mushroom (*Pleurotus* sp.) is highly regarded for its significant contributions to both culinary and medicinal purposes, owing to its rich variety of potent compounds. In recent years, there has been a developing interest in the production of metallic nanoparticles using a more environmentally friendly approach involving various organic materials derived from fungi, particularly *Pleurotus* sp. The oyster mushroom is at the forefront of macrofungi when it comes to producing nanoparticles and their applications. The oyster mushroom ranked first among macrofungi in terms of producing nanoparticles and their subsequent applications (Owaid, 2019).

While selenium is essential as a trace element for maintaining good health and promoting growth, excessive consumption of this element can be hazardous. On the other hand, selenium nanoparticles exhibited several characteristics, including strong durability, biological effectiveness, and little toxicity. Moreover, they showed better absorption over an extended period of supplementation compared to selenium (Kumar and Prasad, 2021). Selenium nanoparticles have been identified as highly promising agents with diverse therapeutic uses. Previous research has documented the antioxidative properties of selenium nanoparticles (Zheng et al., 2020). Selenium nanoparticles have demonstrated antimicrobial capabilities against different human and plant pathogens (Abdelsalam et al., 2023).

Based on the increasing worldwide population, there is a pressing demand for top-notch food to ensure global food security. Conventional fertilizers and insecticides encounter issues related to low efficiency in usage and potential risks to non-target organisms. Consequently, SeNPs have generated significant interest and have been identified as having potential use in agriculture (Song et al., 2023). The potential utilization of SeNPs in agriculture encompasses various benefits such as reducing the impact of both living organisms and environmental factors on plants, enhancing the process of seed germination and overall plant development, as well as enhancing the Selenium levels and nutritional value in crops (Song et al., 2023). Wheat, scientifically known as *Triticum asetivum* L., belongs to the Poaceae family. It is a significant cereal crop and the primary strategic crop in Egypt and globally. In Egypt, the cultivation of grains on irrigated fields resulted in a wheat crop production of approximately 9.2 million tons during the 2019–2020 season (El-Saadony et al., 2021). Nevertheless, Egypt holds the title of being the largest global importer of wheat, as reported by the Food and Agriculture Organization in 2015 and 2018. Wheat is a significant source of energy due to its high content of carbohydrates and protein. In addition, the presence of phytochemicals and dietary fibers in this cereal significantly lowers the chances of developing cardiovascular disease, colon-rectal cancer, and type 2 diabetes. Consequently, it is considered a highly beneficial food for maintaining good health (El-Saadony et al., 2021). This study highlights the importance of using *Pleurotus ostreatus* for the biosynthesis of selenium nanoparticles (SeNPs), a sustainable and eco-friendly approach that enhances nanoparticle stability and bioactivity. *Pleurotus ostreatus* plays a crucial role in SeNPs synthesis by acting as a natural reducing and stabilizing agent, eliminating the need for toxic chemical reagents. The resulting SeNPs demonstrate multifunctional properties, including significant antimicrobial, antiviral, and plant growth-promoting activities, making them promising candidates for applications in medicine, agriculture, and environmental science. By integrating these diverse applications, our research provides valuable insights into the potential of fungal-assisted nanotechnology, advancing its role in sustainable biomedicine and agricultural innovation. Consequently, this study conducted the mycosynthesis of SeNPs using the aqueous extract of the edible mushroom *Pleurotus ostreatus*. The physicochemical characteristics of the synthesized SeNPs were extensively investigated using various analytical techniques. Additionally, the antioxidant, antimicrobial, and antiviral activities of the SeNPs were evaluated, along with their effects on the growth of *Triticum asetivum* (wheat).

## 2 Materials and methods

### 2.1 Fungal strain

The fungal strain *Pleurotus ostreatus* was obtained from the Agriculture Research Center in Cairo, Egypt. The fungus fruiting bodies were first subjected to a drying process, then homogenized into a fine powdered form. This powdered fungal material was preserved in a refrigerator at 4°C for subsequent usage in the experiments.

### 2.2 Extraction of dry fungal tissues

Aqueous extract of the *Pleurotus ostreatus* fungus was prepared using the following method. Five grams of the air-dried fungal tissues were extracted with 100 mL of deionized distilled water at 60°C for 1 h. After the extraction process, the resulting mixture was let to cool, and subsequently, it underwent filtration using Whatman filter paper No. 1. The clarified solution was obtained by centrifuging the filtered extract for 15 min at a speed of 4,000 rpm (Boly et al., 2016).

### 2.3 Selenium nanoparticles (SeNPs) of *P. ostreatus*

The mycosynthesis of selenium nanoparticles (SeNPs) was carried out with some modifications following the procedure stated by Morad et al. (2022). Briefly, 1 gram of sodium selenite was mixed with 50 mL of *Pleurotus ostreatus* fungal extract. The resulting mixture was then left to incubate for a duration of 2 days, maintaining a temperature of 40°C.The formation of the selenium nanoparticles was evident from the change in color of the solution, which turned into a cloudy orange hue. To determine the efficiency of SeNPs synthesis, the following formula was used:


$$\mathrm{Efficiency}\;\left(\%\right)=\frac{\mathrm{Mass of synthesized SeNPs}}{\mathrm{Mass of sodium selenite used}}\times 100$$

### 2.4 Characterization of myco-synthesized selenium

The physicochemical properties of the myco-synthesized selenium nanoparticles (SeNPs) were extensively analyzed using several advanced analytical instruments. These methods included a UV–visible spectrophotometer (UV-2600 spectrophotometer (Shimadzu, Japan)), a Zetasizer analyzer (Malvern Panalytical, United Kingdom), a transmission electron microscope (TEM) using a JEM-2100 microscope (JEOL, Japan), and a Fourier-transform infrared (FTIR) spectrophotometer (Nicolet iS50 FTIR spectrometer (Thermo Fisher Scientific, United States)). The UV–visible spectrophotometer was utilized to examine the optical characteristics of the SeNPs, and the absorbance was measured within the wavelength range of 200–800 nm. This analysis was performed using equipment from PerkinElmer Life and Analytical Science, located in CT, Ohio, United States. A particle size analyzer, specifically the Zetasizer Nano ZN from Malvern Panalytical Ltd. in Malvern, UK, was employed to determine the average diameter size, size distribution, and zeta potential charges of the SeNPs. The DLS analysis was conducted at a fixed angle of 173 degrees and a temperature of 25°C. High-resolution transmission electron microscopy (HR-TEM) using the JEOL 2100 instrument from Japan, equipped with an electron diffraction pattern, was used to capture transmission electron micrographs of the SeNPs. Additionally, a Fourier-transform infrared (FTIR) spectrophotometer from Perkin Elmer in Ohio, USA, was utilized to investigate the elemental structure and identify the functional groups present in the synthesized selenium nanoparticles. All analyses were performed in triplicate to ensure the accuracy and reliability of the results. These comprehensive analytical techniques provided a thorough understanding of the various characteristics of the synthesized selenium nanoparticles.

### 2.5 Total antioxidant activity

The antioxidant activity of the biosynthesized selenium nanoparticles (Se-NPs) was evaluated by assessing their capacity to eliminate DPPH (2,2-diphenyl-1-picrylhydrazyl) radicals. Various concentrations of the SeNPs were tested in a 96-well plate, with each well (*n* = 6) containing 100 μL of freshly prepared DPPH reagent (0.1% in methanol) and 100 μL of the sample. The reaction mixture was then incubated at room temperature for 30 min in the dark. After the incubation period, the reduction in the DPPH color intensity was measured at a wavelength of 540 nm. Trolox, a standard antioxidant compound, was used as the reference. The data is presented as means ± standard deviation (SD), and the results were analyzed according to the following equation (Cittrarasu et al., 2021).


$$\mathrm{Percentage}\;\mathrm{inhibition}=\frac{\begin{array}{l} \mathrm{Average absorbance of blank}-\\ \mathrm{average absorbance of the test} \end{array}}{\mathrm{Average absorbance of blank}}*100$$

### 2.6 *In vitro* antimicrobial activity

The antimicrobial activity of the myco-synthesized selenium nanoparticles (SeNPs) was evaluated against three clinically significant pathogens: *Escherichia coli* (ATCC 25922), *Staphylococcus aureus* (ATCC 25923), and *Candida albicans* (ATCC 1031). These microbial strains were obtained from the Mycology Laboratory, Faculty of Science, Helwan University, Cairo, Egypt. For bacterial strains, nutrient agar (NA) plates were used, while Sabouraud dextrose agar (SDA) plates were used for fungal cultures. The inoculum density was adjusted to 0.5 McFarland standard (~1.5 × 10^8^ CFU/mL) before plating. Wells of 6 mm diameter were made in the agar using a sterile cork borer. 100 μL of SeNP solution (20 μg/mL in sterile distilled water) was added to each well. The plates were pre-incubated at 4°C for 8 h to facilitate diffusion of SeNPs, followed by incubation at 37 ± 2°C for 24 h. The antimicrobial activity was determined by measuring the diameter of inhibition zones (mm), including the well diameter, using a digital caliper. Each test was performed in triplicate, and the average inhibition zone size was recorded. Vancomycin (20 μg/mL) and gentamicin (20 μg/mL) were used as standard antibacterial controls, while amphotericin B (20 μg/mL) served as the antifungal control. A well containing 100 μL of sterile distilled water was used as a negative control. The results were expressed as mean inhibition zone diameters ± standard deviation (SD) (Miglani and Tani-Ishii, 2021).

### 2.7 Antiviral bioactivity evaluation

#### 2.7.1 Cytotoxicity (CC_50_) determination

A low pathogenic coronavirus (229E) was used in this study. The CC_50_ (half-maximal cytotoxic concentration) was calculated to assess the cytotoxicity of the selenium nanoparticles (SeNPs) on Vero E6 cells before conducting the IC50 (half-maximal inhibitory concentration) determination. This step was essential to ensure that the observed antiviral effects of SeNPs were not due to cytotoxicity but rather due to direct inhibition of viral replication. The half-maximal cytotoxic concentration (CC_50_) of the tested compound was determined by first preparing stock solutions in double-distilled water (ddH_2_O), which were then further diluted to working solutions using Dulbecco’s Modified Eagle’s Medium (DMEM). The crystal violet assay was employed to evaluate the cytotoxic activity of the SeNPs on the Vero E6 cell line. In brief, the cells were placed in 96-well plates at a concentration of 3 × 10^5^ cells/ml, with 100 μL in each well, and left to grow for 24 h at a temperature of 37°C in an environment with 5% CO_2_. Following a 24-h period, the cells were subjected to different concentrations of the tested SeNPs in three separate treatments. After a 72-h period of incubation, the liquid portion was removed, and the layers of cells were treated with a 10% solution of formaldehyde for 1 h at room temperature. The formed monolayers were subsequently dried and treated with 50 μL of 0.1% crystal violet solution for 20 min at ambient temperature. Afterward, the monolayers were rinsed, let to dry for the entire night, and the crystal violet dye in each well was dissolved in 200 μL of methanol for 20 min at room temperature. Using a multi-well plate reader, the absorbance of the crystal violet solutions was determined at 570 nm. Using GraphPad Prism software (version 5.01), nonlinear regression analysis was used to get the CC_50_ value. This involved plotting the compound’s log concentrations against the normalized response (AbouAitah et al., 2021).

#### 2.7.2 Half-maximal inhibitory concentration (IC_50_) determination

Using 96-well tissue culture plates, Vero E6 cells (3 × 10^5^) were carefully placed in each well and left to grow overnight in a warm and humid incubator at 37°C with a controlled CO_2_ concentration of 5%. After the cell monolayers were rinsed with 1x phosphate-buffered saline (PBS), they were exposed to a low pathogenic coronavirus (229E) for 1 h at room temperature. Subsequently, the cell monolayers were covered with 50 μL of Dulbecco’s Modified Eagle’s Medium (DMEM) that contained different concentrations of SeNPs. Following a 72-h period of growth at a temperature of 37°C in an incubator containing 5% CO_2_, the cells were treated with 100 μL of a 4% solution of paraformaldehyde for 20 min. Subsequently, the cells were exposed to a 0.1% staining solution of crystal violet in distilled water for 15 min at room temperature. The crystal violet dye was subsequently dissolved by adding 100 μL of 100% methanol each well, and the intensity of the color was quantified at a wavelength of 570 nm using an Anthos plate reader (BMGLABTECH®FLUOstar Omega, Germany). The IC_50_ of SeNPs refers to the concentration at which the viral-induced cytopathic effect (CPE) is reduced by 50% compared to the virus control (AbouAitah et al., 2021).

### 2.8 Effect of nano-selenium on *T. aestivum* growth

Wheat (*Triticum aestivum*) seeds were obtained from the Agriculture Research Center, Cairo, Egypt. Healthy and uniform seeds were surface-sterilized by soaking in 70% ethanol for 2 min, followed by three washes with sterile distilled water to remove any contaminants. The sterilized seeds were soaked in selenium nanoparticles (SeNPs) solutions at different concentrations (0, 1, 5, 10, 25, 50, and 100 μM) for 12 h to ensure adequate absorption. After soaking, the seeds were sown in small plastic pots (10 cm diameter) containing loamy soil and placed in a controlled growth chamber at the Plant Physiology Laboratory, Faculty of Science, Helwan University. The pots were maintained at 70% field capacity with regular irrigation using tap water. After 15 days of growth, plant samples were collected for morphological and biochemical analysis. Growth parameters, including shoot length, root length, and biomass production, were recorded using a digital caliper and precision balance. The biochemical analysis included the determination of total soluble sugars and total soluble protein content, measured spectrophotometrically using standard protocols. All experiments were conducted in triplicate, and results were expressed as mean ± standard deviation (SD) (Zafar et al., 2024).

#### 2.8.1 Total soluble sugars

Soluble sugars were extracted from 0.1 g of freshly collected leaves using 5 mL of 70% ethanol. The supernatant was subsequently dehydrated by centrifugation until it attained a predetermined volume. The anthrone protocol, as outlined by Umbriet et al. (1959), was employed to determine the overall sugar content. Three milliliters of an anthrone solution, which contains 2 g per liter of 95% H_2_SO_4_, were mixed with 1 m of a plant extract sample. Next, the resultant mixture was immersed in a boiling water bath for a period of 3 min. The color produced was quantified at a wavelength of 620 nm using spectrophotometry after the completion of temperature lowering.

#### 2.8.2 Total soluble proteins

A quantity of 0.1 g of freshly harvested leaves was extracted using 5 mL of 70% ethanol. The supernatant was then centrifuged until it reached a known volume. To quantify the soluble protein content, a new solution consisting of 5 mL of a 2% solution of sodium carbonate in 0.4% sodium hydroxide and 0.5% copper sulphate in 1% sodium tartrate was mixed with 1 mL of extract. The volume (v/v) ratio was 50:1. Following a ten-minute reaction period, the mixture was adjusted to a precise volume by introducing 0.5 mL of Folin phenol reagent in a 1:1 ratio. After 30 min, the optical density of the combination was measured at 750 nm (Lowry et al., 1951).

### 2.9 Volatile compounds detection by gas chromatography–mass spectrometry (GC–MS) technique

An Agilent 7,000 Series Triple Quad Gas Chromatograph paired with a mass spectrometer was used to examine the active metabolites present in the fungal extract. The analysis was conducted using the following conditions: The analytical setup included a capillary column with dimensions of 30 m length, 0.25 m internal diameter, and 0.25 df film thickness. The electron ionization was performed at an energy level of 70 electron volts. The carrier gas used is 99.999% pure helium, flowing at a rate of 1 milliliter per minute. The injector temperature was set at 250°C, and a volume of 1 microliter is injected. The injection is split at a ratio of 1:10. Lastly, the ion-source temperature was maintained at 200°C. The oven was initially heated to 110°C and maintained at that temperature for 2 min. Then, the temperature was increased at a rate of 10°C per minute until it reached 200°C. After that, the temperature was further increased at a rate of 5°C per minute until it reached 280°C. Finally, the temperature was held at 280°C for a duration of 9 min. The spectral mass was obtained at an energy level of 70 electron volts (eV). The scan interval was 0.5 s, and the fragment sizes ranged from 45 to 450 Daltons (Da). The entire duration of the gas chromatography (GC) run was 36 min. The relative proportion of each component was obtained by comparing the peak area of the component to the total peak area. The spectrum data and chromatogram peaks were examined using the Turbomass software to determine the volatile compounds found in the fungal extract (Mulatu et al., 2022).

### 2.10 Docking study

Molecular docking of SeNPs was conducted against seven protein targets - the *E. coli* multidrug efflux pump AcrB (4DX5), *S. aureus* efflux pump MmpL3 (7LO8), *C. albicans* sterol 14α-demethylase CYP51 (5V5Z), human peroxiredoxin-5 (1HD2) to study its antioxidant effects, SARS-CoV-2 spike protein (7QUS), photosystem II protein D1 (P35860), and gibberellin receptor GA receptor GID1 (2ZSH) to study the effect on plant enhancement of growth using docking algorithm in MOE (Molecular Operating Environment 2014.09). Protein structures were prepared by adding hydrogens, adjusting protonation states and calculating partial charges with Amber10: EHT force field. SeNPs structure in mol2 format was obtained from PubChem. Rigid protein-flexible ligand docking was performed with scoring of top poses based on binding affinity (ΔG binding), hydrogen bonding and interactions at the binding interface. Key residue interactions and binding motifs were analyzed to provide insights into potential interaction mechanisms between SeNPs and these targets relating to their known biological functions.

### 2.11 Statistical analysis

Each experimental result was shown as the mean of three replicates with a standard deviation. An examination of statistical significance was performed comparing data with IBM SPSS Statistics 21 software. A one-way ANOVA and Duncan’s multiple range analysis were conducted at a level of significance of *p* < 0.05.

## 3 Results

### 3.1 Synthesis of myco-synthesized selenium

**Figure 1:**
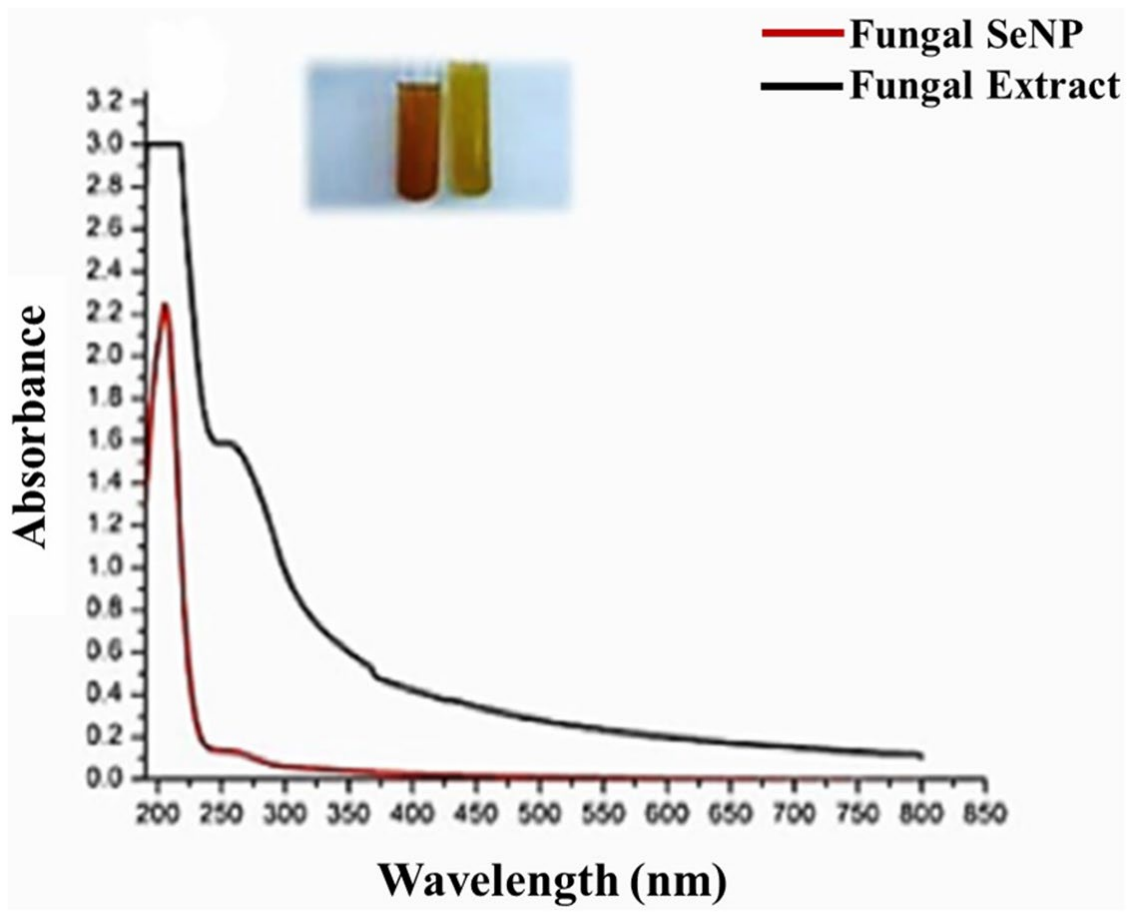
UV–visible absorption spectrum and the red color of myco-synthesized SeNPs after 24 h.

SeNPs were successfully synthesized from aqueous extracts of *Pleurotus ostreatus* after 24 h. The color of the culture medium underwent a noticeable change, shifting from yellow to ruby red. This transformation occurred when the culture filtrate exposed to 1 mM sodium selenite, as shown in Figure 1. The transformation of color from yellow to ruby red indicated the formation of SeNPs. The process of SeNPs formation was confirmed after incubation period that showed the presence of ruby red color within the culture medium which served as compelling evidence that the extracellular metabolites converted selenite ions into the elemental selenium form. The productivity of SeNPs was quantified as approximately 12.5 mg per 100 mL of culture medium. Based on the initial sodium selenite concentration, the nanoparticle synthesis efficiency was calculated to be 0.625%. This demonstrates the efficiency of myco-synthesis method in producing nanoparticles.

### 3.2 Characterization of myco-synthesized selenium

**Figure 2:**
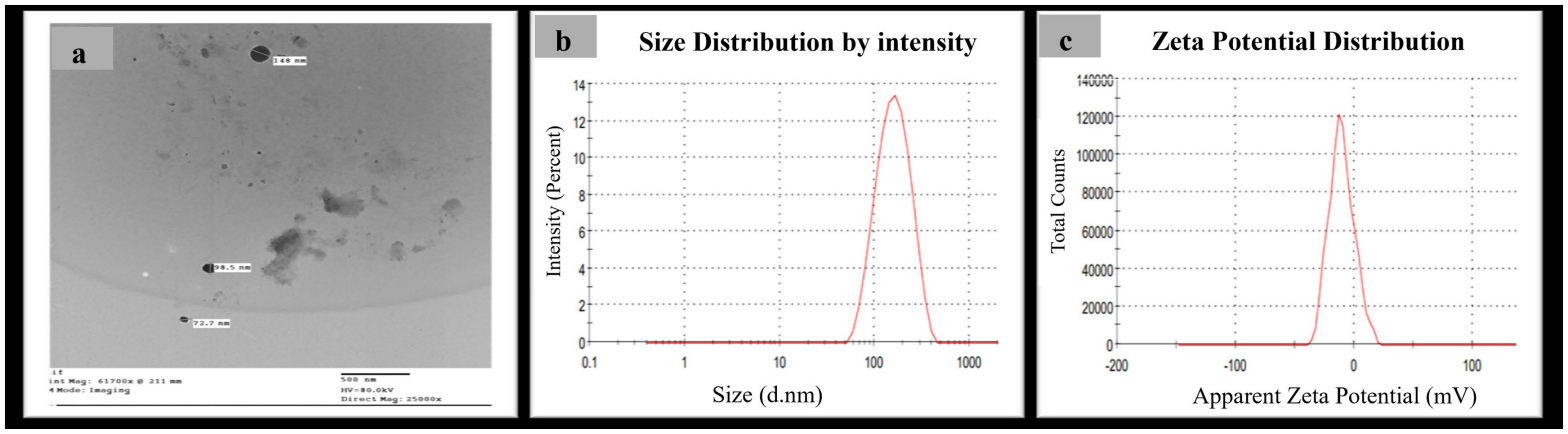
Morphological characterization of myco-synthesized SeNPs **(a)** TEM photographs of myco-synthesized SeNPs using *Pleurotus ostreatus* aqueous extract at scale of 500 nm and magnification 2,500X, **(b)** size distribution pattern, and **(c)** zeta potential distribution.

The production of the synthesized SeNPs was meticulously tracked using a UV–visible spectrophotometer, which revealed a prominent and high surface plasmon resonance (SPR) peak at 205 nm (Figure 1), a clear indication of their successful formation, in contrast to the control sample where no such peak was observed. Further analysis using transmission electron microscopy (TEM) confirmed the identity of the SeNPs, exhibiting a spherical morphology with a diameter size range of 72–148 nm (Figure 2a), while dynamic light scattering (DLS) measurements determined the average diameter of the SeNPs synthesized through the mycological method to be 153 nm (Figure 2b), with the size being influenced not only by their metallic core but also by the biomolecules that coat their surfaces, serving as stabilizers. The stability of these SeNPs was evaluated by assessing their zeta potential, which revealed a mean value of approximately −10.5 mV (Figure 2c), indicating stability of the colloidal system. The homogeneity or heterogeneity of the colloidal nanoparticles was evaluated employing the polydispersity index (PDI) value. In the present research, the PDI result was 0.27. Collectively, these analytical techniques provided a comprehensive understanding of the physical characteristics and stability of the SeNPs synthesized through the efficient mycological approach.

**Figure 3:**
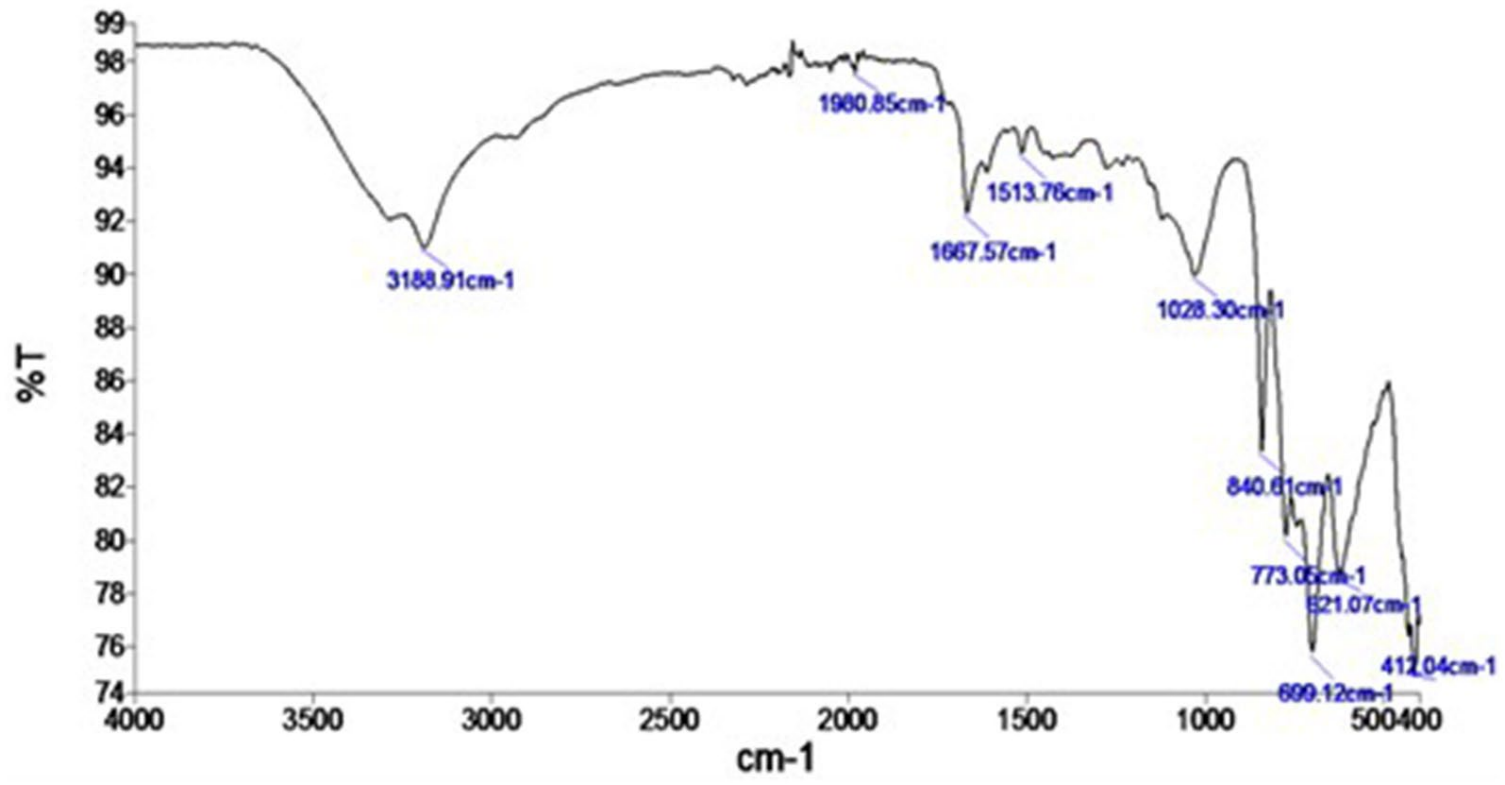
FTIR pattern of myco-synthesized selenium nanoparticles, where Y-axis represented the transmission (% T) and X-axis represented the wavelength (cm ^−1^).

The FTIR analysis of the synthesized SeNPs revealed information about the functional groups present in the metabolites responsible for their myco-synthesis and stabilization. A total of ten prominent peaks were observed, with the wide band at 3188.91 cm^−1^ attributed to the N-H stretching vibrations of amide A in proteins, the peak at 1980.85 cm^−1^ corresponding to the C=C stretching vibrations of alkenes, the distinct and intense narrow peak at 1667.57 cm^−1^ identified as the amide I band characteristic of protein backbones, the peak at 1513.76 cm^−1^ assigned to the amide II band, the peak at 1028.30 cm-1 attributed to the C-N stretching vibrations, the peaks at 840.61, 773.05, 699.12, and 621.07 cm^−1^ identified as C-X stretches indicating the presence of halogenated compounds, and the peak at 412.04 cm^−1^ assigned to the C-N-C stretching vibrations. Collectively, these metabolites responsible for the efficient myco-synthesis and stabilization of the synthesized SeNPs (Figure 3).

### 3.3 Biological activity evaluation

#### 3.3.1 Total antioxidant activity

The antioxidant efficacy of the formulated SeNPs was comprehensively evaluated by comparing their DPPH radical scavenging ability to the potent antioxidant, Trolox. As the concentration of the SeNPs increased, their DPPH radical inhibition capacity rose correspondingly, displaying their impressive free radical scavenging properties (Table 1). Notably, the SeNPs exhibited an IC_50_ value of 662.1 ± 1.05 μg/mL while the SeNPs demonstrated, a slightly lower antioxidant activity compared to Trolox, which had an IC_50_ of 24.42 ± 0.87 μM.

**Table 1:** Antioxidant activity of biosynthesized SeNPs.

	IC_50_ (Mean ± SE)
Mycosynthesized SeNPs	662.1 ± 1.05 μg/mL^b^
Trolox	24.42 ± 0.87 μM^a^

The results were expressed as mean ± SE from three separate replicates. Statistical significance was indicated at *p* < 0.05. Means within the same column denoted by different letters exhibit significant differences (*p* < 0.05).

#### 3.3.2 *In-vitro* antimicrobial activity

The antimicrobial potential of the synthesized SeNPs was comprehensively investigated against a diverse panel of medically relevant microorganisms, including *S. aureus*, *E. coli*, and *C. albicans* (Table 2). SeNPs exhibited considerable antimicrobial efficacy against *S. aureus* (17 ± 0.02 mm), followed by *E. coli* (16 ± 1.04 mm), and *C. albicans* (12 ± 0.3 mm) in comparison to the evaluated conventional antibiotics.

**Table 2:** Antimicrobial activity (inhibition zones) of selenium nanoparticles (SeNPs) against selected microbial strains.

Sample	Inhibition zone diameter (mm)		
	*S. aureus ATCC 25923*	*E. coli ATCC 25922*	*C. albicans ATCC 1031*
Fungal SeNPs	17 ± 0.02^a^	16 ± 1.04^b^	12 ± 0.3^b^
Vancomycin (20 μg/mL)	16 ± 0.1^b^	Nt	Nt
Gentamicin (20 μg/mL)	Nt	18 ± 0.1^a^	Nt
Amphotericin B (20 μg/mL)	Nt	Nt	14 ± 0.3^a^

The results for the area of inhibition zone, determined as the diameter of the clear zone surrounding the well, were expressed as means ± SD from three replicates. Means within the same column denoted by different letters exhibit significant differences (*p* < 0.05). The diameter of the well is 5 mm. Nt: not tested.

#### 3.3.3 *In-vitro* antiviral activity

**Figure 4:**
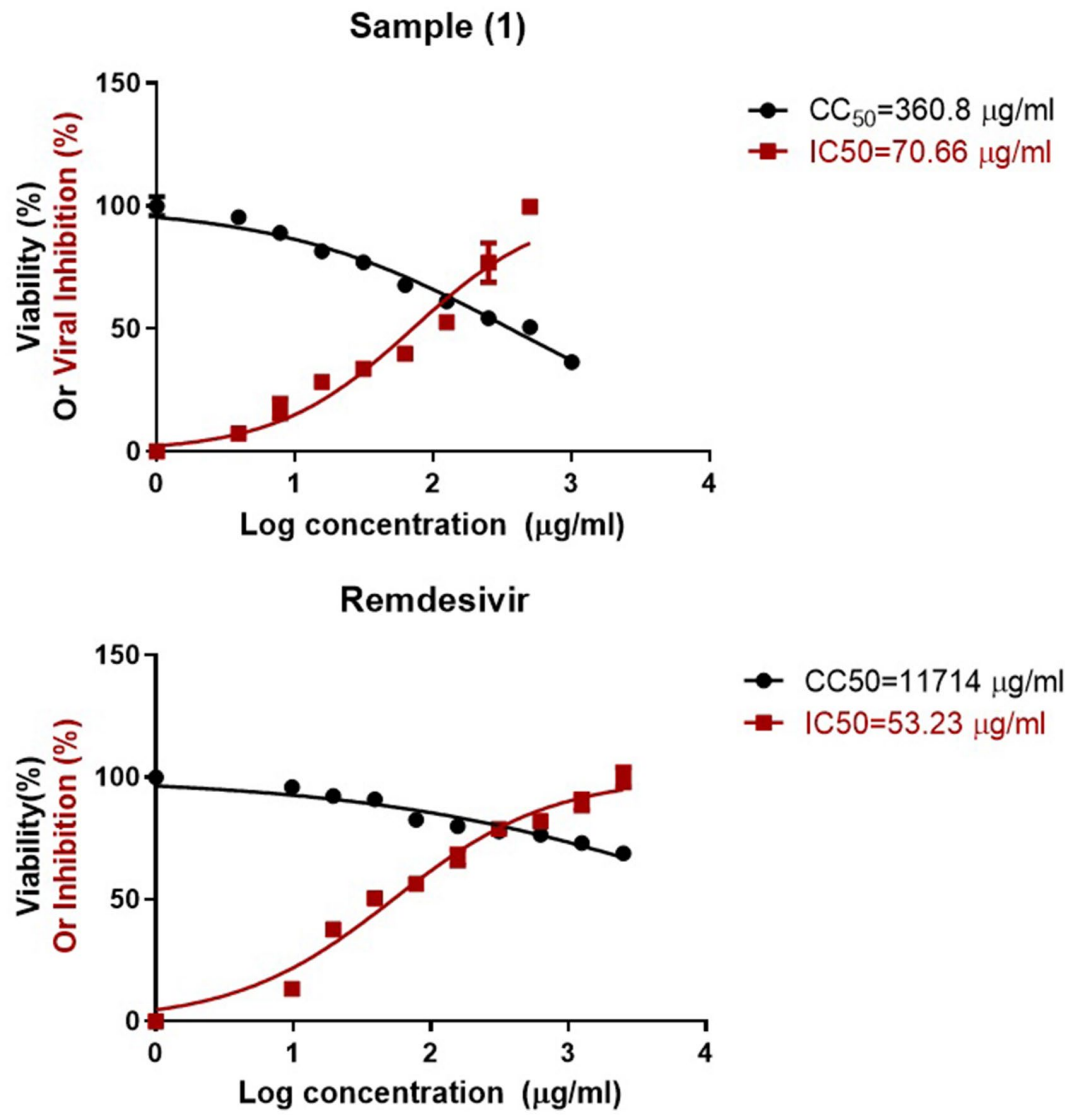
Antiviral activity of biosynthesized SeNPs.

The biosynthesized selenium nanoparticles (SeNPs) exhibited moderate antiviral activity against the low pathogenic coronavirus (229E) strain, with a selectivity index (SI) of 5 calculated as the ratio of the SeNPs’ cytotoxic concentration (CC_50_ = 360.8 μg/mL) to their half-maximal inhibitory concentration (IC_50_); in comparison, the positive control, Remdesivir, demonstrated significantly superior antiviral potency, with a much greater CC_50_ of 11,714 μg/mL on Vero E6 cells and a notably higher SI of 220 (Figure 4).

#### 3.3.4 Effect of nano-selenium on *T. aestivum* growth

**Figure 5:**
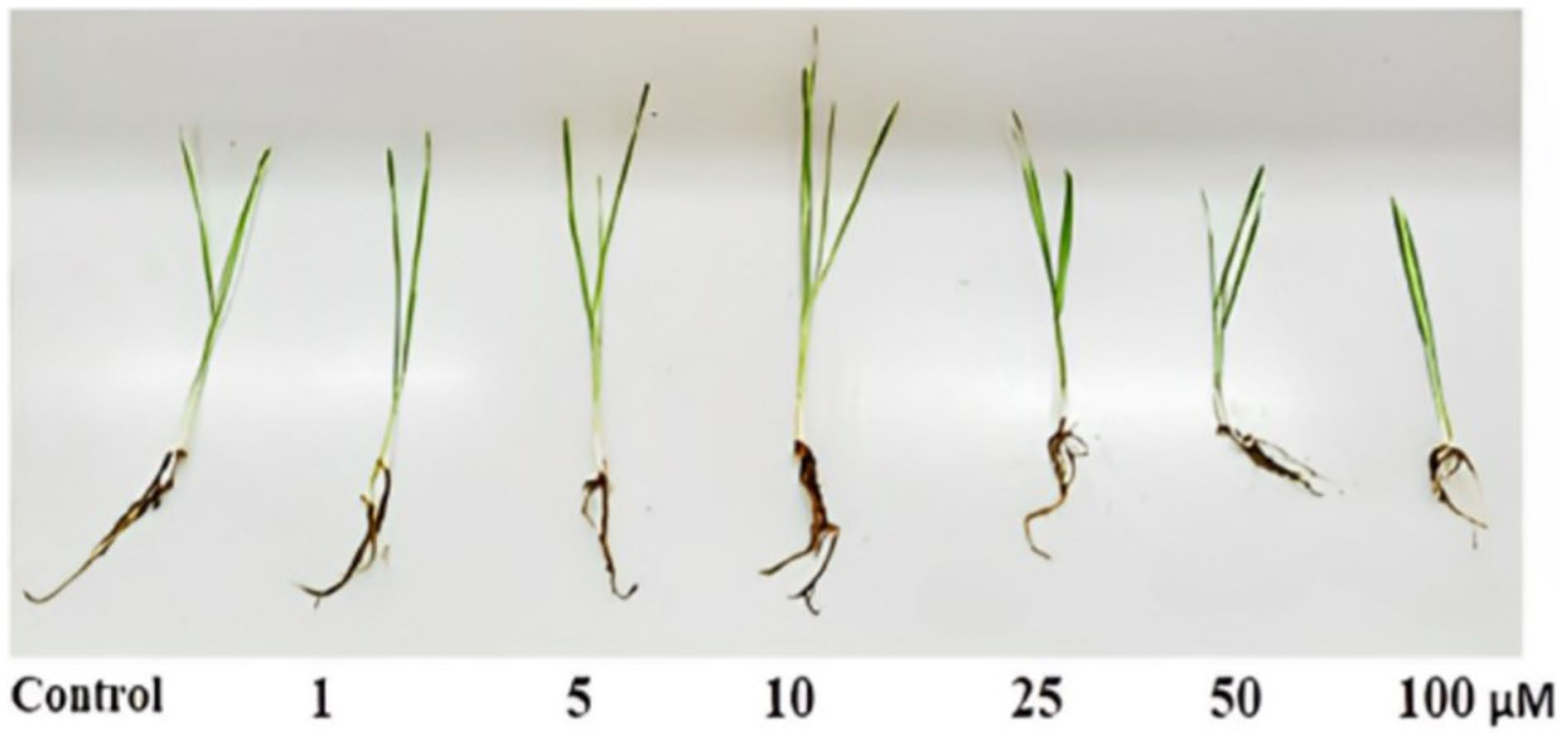
Effect of nano-selenium on *Triticum aestivum* growth.

Selenium nanoparticles effectively promoted *T. aestivum* growth at the three tested concentrations with the most effective concentration at 10 μM compared to the control (Table 3 and Figure 5). Application of SeNPs at dose (10 μM concentration) resulted in a significant increase in primary metabolites, including total soluble sugars (54.32 mg/g) and proteins (139.66 mg/g), compared to the control.

**Table 3:** Effect of selenium nanoparticles (SeNPs) on the growth parameters and biochemical composition of *Triticum aestivum* after 15 days of treatment.

SeNP concentration (μM)	Shoot length (cm)	Root length (cm)	Total soluble sugars (mg/g FW)	Total soluble proteins (mg/g FW)
Control	18.0^cd^	7.0^b^	32.55^c^	126.58^b^
1	19.0^c^	7.0^b^	40.27^b^	130.75^ab^
5	21.0^b^	8.0^a^	41.28^b^	132.87^ab^
10	23.5^a^	8.5^ab^	54.32^a^	139.66^a^
25	17.0^de^	6.3^bc^	30.00^c^	125.47^b^
50	15.5^ef^	5.2^c^	28.7**5**^d^	114.90^c^
100	14.0^e^	5.0^c^	28.1**1**^d^	102.70^b^
LSD: at 0.05	2	1.5	1.3	11.68

Statistical analysis was carried out using LSD and Duncan. Different letters showed significant variation at *p* < 0.05. FW = Fresh Weight.

### 3.4 Detection of volatile compounds by GC–MS technique

**Figure 6:**
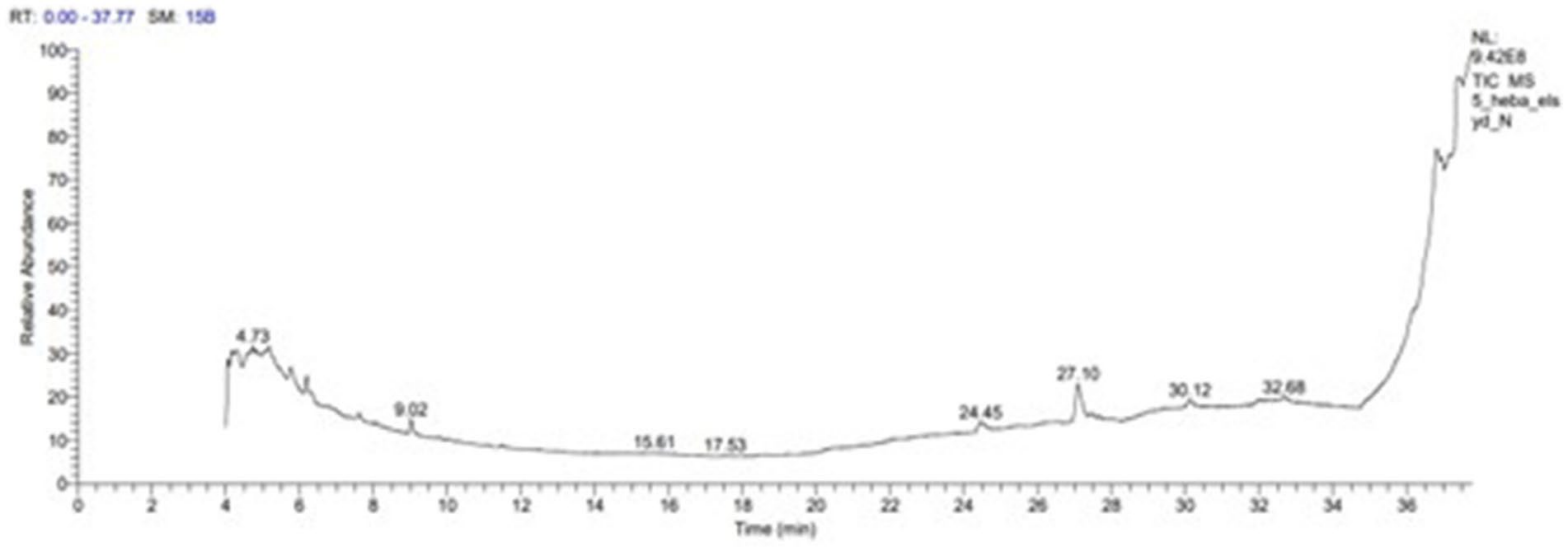
GC- MS of *Pleurotus ostreatus* extract.

The extract of the *P. ostreatus* mushroom underwent gas chromatography–mass spectrometry (GC–MS) (Agilent 7,000 Series Triple Quad, Agilent Technologies, USA) analysis to determine the chemical constituents that contribute to synthesis of SeNPs, detecting the presence of 10 bioactive compounds, with R-Limonene as the predominant component (37.34%) and minor components such as 4 cyclopentene-1,3-diol-D2, trans (10.56%), 1,2,4-cyclopentanetriol (2.33%), 3-(Prop-2-enoyloxy) dodecane (1.44%), and 9,12-Octadecadienoyl chloride, (Z,Z)- (21.69%), among others, several of which have been documented to exhibit antimicrobial, antioxidant, and therapeutic properties, such as 4 cyclopentene-1,3-diol-D2, trans and 4-cyclopentene-1,3-diol, trans- demonstrating antimicrobial activity, and 1,2,4-cyclopentanetriol and 9,12-Octadecadienoyl chloride, (Z,Z)- showing antioxidant and anticancer activities (Table 4 and Figure 6).

**Table 4:** GC- MS of *Pleurotus ostreatus* extract.

Compound name	Molecular formula	Molecular weight	RT (min)	Area sum%	Compound class	Biological activity
4 cyclopentene-1,3-diol-D2, trans	C5H6D2O2	102	4.06	10.56	Cycloalkenes	Antimicrobial activity (Hassan et al., 2023; Hassan et al., 2023)
1,2,4-cyclopentanetriol	C5H10O3	118	4.57	2.33	Cyclic polyol	Antioxidant and Antimicrobial activity (Yassin et al., 2022)
3-(Prop-2-enoyloxy) dodecane	C15H28O2	240	4.64	1.44	Fatty acid esters	Antibiotics (Karthik et al., 2023)
3,4,4-D3-trans-3,5-dihydroxy cyclopentene	C5H5D3O2	103	4.72	2.69	Cyclic hydrocarbon	–
2,4- Pyridine dicarboxylic acid, dimethyl ester	C9H9NO4	195	517	5.40	Aliphatic hydrocarbon	Anticancer activity (Brewitz et al., 2020)
4-Cyclopentene-1,3-diol, trans-	C5H8O2	100	5.76	3.09	monoacetate	Anti-microbial activity (Hassan et al., 2023)
1,3-hexandiol, 2-ehthyl	C8H18O2	146	9.02	2.42	Aliphatic alcohol	Therapeutic effect (Khalid et al., 2022)
9-Octadecenoic acid (Z)	C18H34O2	282	27.09	8.21	Fatty acid	Anti-microbial activity (Zahara et al., 2022)
9,12-Octadecadienoyl chloride, (Z,Z)	C18H31ClO	298	36.80	21.69	Alkyl chloride	Anti-oxidant, anti-cancer and thyroid inhibitor (Khalid et al., 2022)
R-Limonene	C10H16O3	184	37.34	14.78	monoterpene	anti-inflammatory and antioxidant actions (Santana et al., 2020)

### 3.5 Docking study

**Figure 7:**
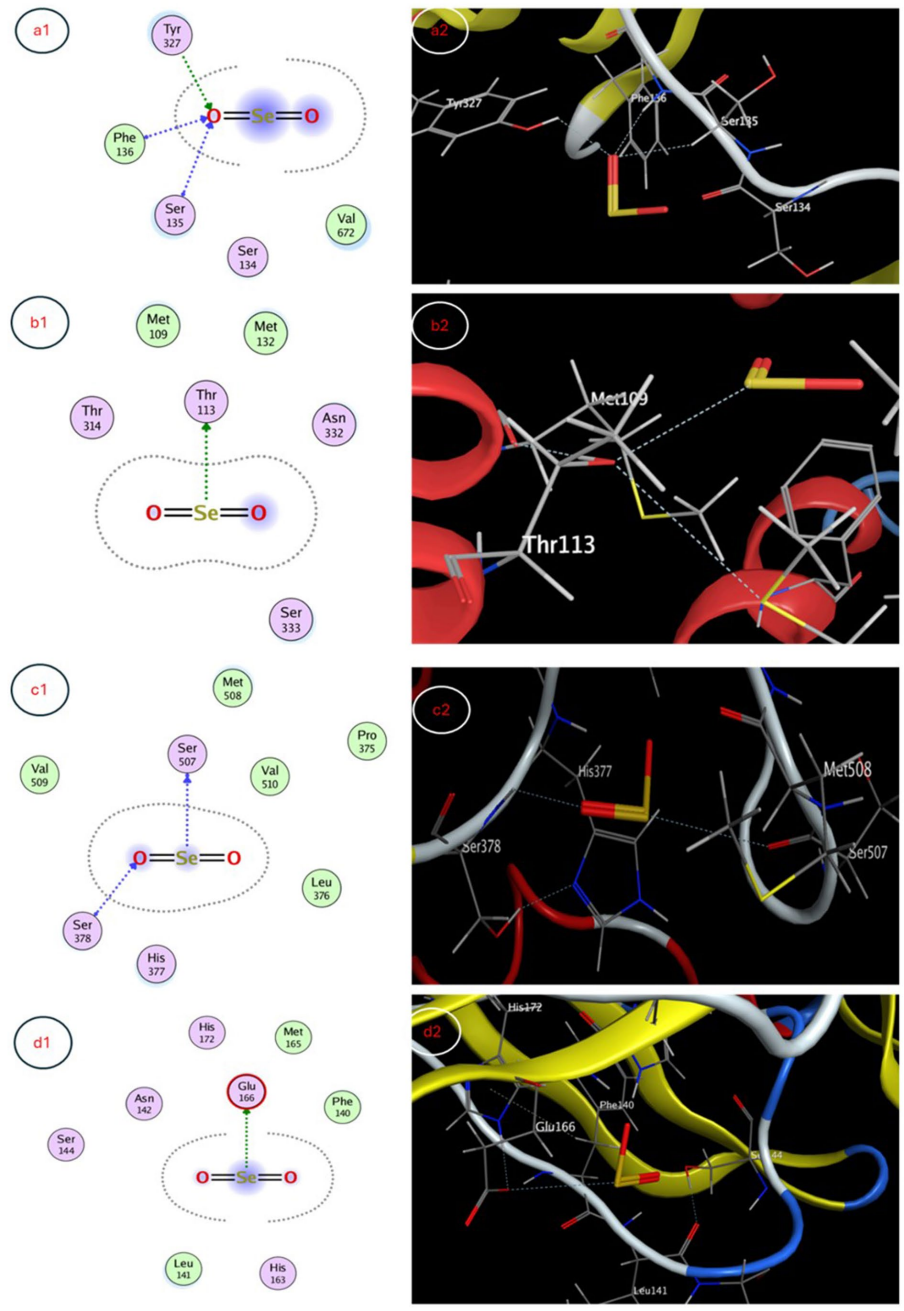
3D and 2D interaction maps depicting favorable docked poses of SeNPs within the active sites of **(a1)** *E. coli* efflux pump AcrB, **(b1)** *S. aureus* efflux pump MmpL3, **(c1)** *C. albicans* sterol 14α-demethylase CYP51 and **(d1)** SARS-CoV-2 spike protein, providing visualization of putative multi-target binding modes.

**Figure 8:**
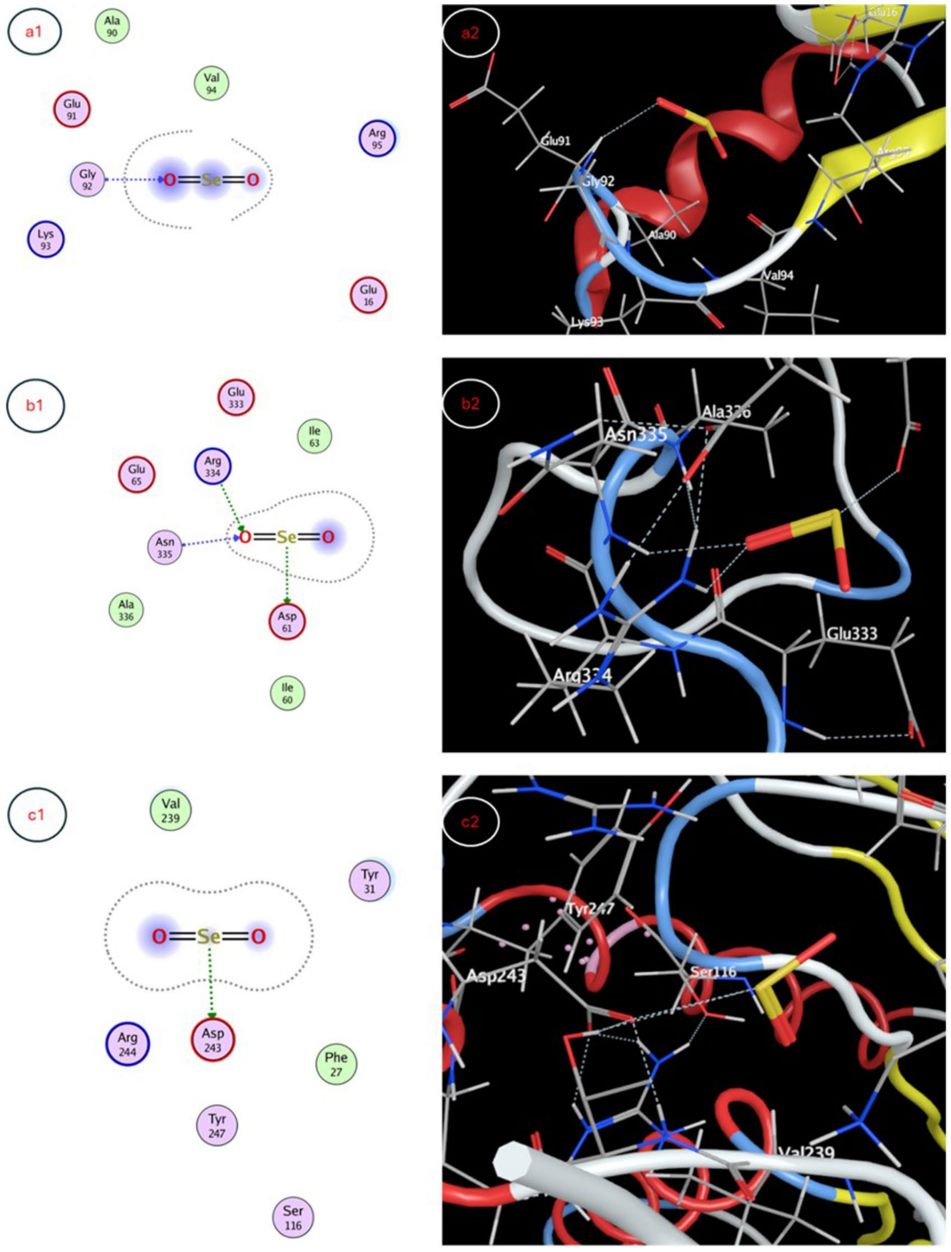
Molecular docking interaction maps depicting SeNP docking poses within active sites of **(a1)** human peroxiredoxin-5, **(b1)** photosystem II protein D1, and **(c1)** gibberellin receptor GA receptor GID1, offering insight into pleiotropic effects of SeNPs through diverse biomolecular interactions.

The molecular docking study evaluated binding of selenium nanoparticles (SeNPs) to seven biologically relevant protein targets. The conformation that exhibited the highest energetic favorability for AcrB (4DX5) revealed interactions between SeNPs and Tyr327 as well as Phe136/Ser135 through hydrogen bonding and hydrophobic contacts, resulting in a binding affinity of −3.4873 kcal/mol. MmpL3 (7LO8) exhibited a score of −3.2825 kcal/mol involving side chain donor Thr113. CYP51 (5V5Z) positioning revealed interactions with Ser507 and Ser378 backbones at −3.4827 kcal/mol. Human peroxiredoxin-5 (1HD2) posed SeNPs at −3.0635 kcal/mol near Gly92. SARS-CoV-2 spike protein (7QUS) favored liaisons to Lys-A1028 and Phe-A1042 with a score of −3.2556 kcal/mol. Photosystem II D1 (P35860) pointed to Arg334, Asp61 and Asn335 recognizing SeNPs at −3.1927 kcal/mol. The GA receptor GID1 (2ZSH) complex placed SeNPs near Asp243 with −3.1778 kcal/mol binding potential. This *in silico* analysis identified putative binding motifs that may infer how SeNPs engage these molecular targets (Table 5, Figures 7, 8).

**Table 5:** Molecular docking interaction maps of SeNPs depicting binding poses within active sites of targets modulating biological processes.

Protein target	Docking score (Kcal/mol)	Interaction type	Amino acid residue
*Escherichia coli* efflux pump AcrB	−3.4873	Side chain acceptor	Tyr 327
		Backbone acceptor	Phe 136
			Ser 135
*Staphylococcus aureus* efflux pump MmpL3	−3.2825	Side chain donor	Thr 113
*Candida albicans* sterol 14α-demethylase CYP51	−3.4827	Backbone donor	Ser 507
		Backbone acceptor	Ser 378
SARS-CoV-2 spike protein	−3.0635	Backbone acceptor	Gly 92
Human peroxiredoxin-5	−3.2556	Side chain acceptor	Lys-A 1028
		Backbone acceptor	Phe-A 1042
Photosystem II protein D1	−3.1927	Side chain acceptor	Arg 334
		Side chain donor	Asp 61
		Backbone acceptor	Asn 335
Gibberellin receptor GA receptor GID1	−3.1778	Side chain acceptor	Asp 243

## 4 Discussion

The synthesis of nanoparticles (NPs) from biomaterials has gained significant attention due to their distinctive attributes, including affordability, straightforward production methods, high water solubility, and eco-friendly characteristics. Macrofungi, particularly diverse mushroom species like *Pleurotus* spp., have emerged as promising sources for NP synthesis, as they possess a wealth of beneficial properties. These mushrooms are renowned for their high nutritional value, immune-modulating effects, and a broad spectrum of bioactivities, encompassing antimicrobial (antibacterial, antifungal, and antiviral), antioxidant, and anticancer properties. The exploration of mushroom-derived NPs holds immense promise, as they can be leveraged to develop multifunctional materials with diverse applications in pharmaceuticals, biomedicine, and environmental remediation, capitalizing on the inherent advantages of both nanoparticles and macrofungal biomaterials (Bhatt et al., 2023).

This study efficiently produced selenium nanoparticles (SeNPs) utilizing aqueous extract derived from the *P. ostreatus* mushroom within 24 h. These results were in agreement with Bhardwaj et al. (2020) who reviewed that two methods have been used to synthesize metal NPs from macrofungi, both the intracellular and extracellular methods.

The red coloration of the reaction mixture was attributed to the excitation of Plasmon vibrations of the SeNPs, providing a distinctive spectroscopic signature of their formation, consistent with findings from other studies (Kanchi et al., 2020; Zambonino et al., 2021). The UV–visible spectra exhibited a broad peak at 205 nm, indicating the production of SeNPs, with the broadness of the peak suggesting the synthesized particles were polydisperse (Piacenza et al., 2024).

Transmission electron microscopy (TEM) analysis revealed the SeNPs had a spherical morphology with a diameter size range of 72–148 nm, within the reported size range for SeNPs (Kanchi et al., 2020). Dynamic light scattering (DLS) studies indicated that the biosynthesized particles had an average diameter of 153 nm, slightly larger than the Transmission Electron Microscopy (TEM) results, likely due to the DLS technique measuring the hydrodynamic diameter, which includes the particle core and any surrounding solvation layer or adsorbed molecules (Tripathi et al., 2020).

The zeta potential of the biosynthesized SeNPs was recorded at −10.5 mV, indicating a negative charge nature. This negative zeta potential suggests the nanoparticles possess a level of stability in the colloidal solution, aided by repulsive forces that deter aggregation to some extent (Shrestha et al., 2020).

The biosynthesis of selenium nanoparticles (SeNPs) using *Pleurotus ostreatus* extract is facilitated by diverse bioactive compounds, including polysaccharides, proteins, phenolic compounds, and flavonoids, which act as natural reducing and stabilizing agents. During this process, selenium ions (SeO₃^2−^) are reduced to elemental selenium (Se^0^) through enzymatic and non-enzymatic pathways. Enzymes such as nitrate reductase and glutathione reductase contribute to the reduction of SeO₃^2−^, while phenolic compounds and flavonoids provide additional electron-donating capabilities, enhancing the stability and controlled growth of SeNPs (Zambonino et al., 2021). The presence of proteins and polysaccharides facilitates nanoparticle stabilization, preventing aggregation and improving colloidal stability (Nayak et al., 2021). The bimolecular capping of SeNPs further enhances their biocompatibility, making them effective for biological applications (Saravanan et al., 2021).

Mushrooms serve as a rich reservoir of potent antioxidant compounds, including phenolics and ergothioneine, which play a crucial role in living systems by mitigating the harmful effects of oxidative stress. These antioxidant molecules function by counteracting the deleterious impact of free radicals, which are produced during normal metabolic processes within the body. Antioxidants contribute to general well-being and may lower the risk of oxidative stress-related disorders by scavenging these free radicals and preserving cellular integrity and health (Goyal et al., 2025).

This study assessed the antioxidant capacity of biosynthesized selenium nanoparticles (SeNPs) utilizing the DPPH assay. The results revealed that the SeNPs exhibited an IC_50_ value of 662.1 ± 1.05 μg/mL, while the fungal extract had an IC_50_ of 800.9 ± 1.04 μg/mL. Although the SeNPs demonstrated moderate antioxidant activity compared to the fungal extract and the positive control, Trolox, these findings indicate that the myco-synthesized selenium nanoparticles function as efficient radical scavengers and possess noteworthy antioxidant properties (Kanchi et al., 2020; Sentkowska and Pyrzyńska, 2023). This observation might be related to the fact that the fungal extract acts as a more robust oxidizing agent, with a greater capacity to induce oxidation and lose electrons more readily compared to the SeNPs (Devi et al., 2023).

The multifunctionality of SeNPs, demonstrated in this study, highlights their distinct roles as antimicrobial agents and plant growth enhancers, each contributing uniquely to environmental sustainability. The biosynthesized selenium nanoparticles (SeNPs) exhibited significant antimicrobial activity against *S. aureus* ATCC25923 (17 mm), *E. coli* ATCC25922 (16 mm), and *C. albicans* ATCC1031 (12 mm), demonstrating greater efficacy to Gram positive bacteria than Gram negative bacteria and fungi, which aligns with previous findings (Pang et al., 2016; Kanchi et al., 2020) linking SeNPs’ potent antimicrobial properties to their diminutive size, large surface area, and capacity to disrupt microbial cell structures and metabolic processes; the variation in inhibition across bacterial and fungal strains is likely due to differences in cell wall composition, where the thick peptidoglycan layer in *S. aureus* enhances nanoparticle penetration, whereas the outer membrane of *E. coli* and the chitin-rich cell wall of *C. albicans* act as partial barriers, although SeNPs’ ability to induce oxidative stress and membrane disruption still contributes to their antimicrobial effects, underscoring their potential for medical, food preservation, and environmental applications while warranting further investigation into their precise mechanisms of action (Filipović et al., 2021). Several studies have highlighted the potential of macrofungi in nanoparticle biosynthesis. For instance, *Ganoderma applanatum* was used to synthesize gold nanoparticles with significant antioxidant and anticancer properties (Abdul-Hadi et al., 2020). Similarly, *Aspergillus terreus* and *Fusarium oxysporum* have been reported to produce selenium nanoparticles with notable antimicrobial activity (Saied et al., 2023).This aligns with studies by Hassanisaadi et al. (2023), where *Aspergillus niger*-derived SeNPs showed similar antimicrobial potential. The antiviral activity of SeNPs is primarily attributed to their ability to interact with viral surface proteins, inhibit viral replication, and modulate immune responses. Studies suggest that SeNPs can bind to viral envelope proteins, preventing host cell attachment and entry (Kopel et al., 2022). Additionally, SeNPs may interfere with viral RNA polymerase activity, disrupting viral genome replication. Selenium’s known immunomodulatory properties also contribute to antiviral defense by enhancing T-cell proliferation and cytokine regulation (Sarkar et al., 2023). In agriculture, SeNPs have been recognized as bio-stimulants that enhance nutrient uptake, stress tolerance, and hormonal regulation. SeNPs improve plant resilience to abiotic stressors such as drought and heavy metal toxicity by modulating the activity of antioxidant enzymes, including catalase and peroxidase (Song et al., 2023). Furthermore, SeNPs influence plant hormonal pathways, particularly gibberellin signaling, promoting seed germination and root elongation (Zafar et al., 2024).

The antimicrobial efficacy of SeNPs is attributed to multiple mechanisms, including cell membrane disruption, protein oxidation, and DNA damage. SeNPs generate reactive oxygen species (ROS), such as hydrogen peroxide (H₂O₂) and superoxide anions, which induce oxidative stress in microbial cells, leading to lipid peroxidation and membrane destabilization (Nowruzi et al., 2023). Additionally, SeNPs interfere with bacterial enzymatic pathways by binding to thiol (-SH) groups in key metabolic proteins, ultimately leading to microbial cell death (Bhardwaj et al., 2020). SeNPs also exhibit potent antioxidant properties by scavenging free radicals and enhancing cellular antioxidant defenses. Selenium, as an essential cofactor for glutathione peroxidase, plays a crucial role in neutralizing oxidative stress (Santana et al., 2020). The interaction of SeNPs with redox-active proteins helps modulate oxidative damage in cells, reducing inflammation and promoting cellular homeostasis (Zeeshan et al., 2022).

Simultaneously, SeNPs play a crucial role in promoting plant growth, as evidenced by their positive effects on *T. aestivum* (wheat). Unlike conventional fertilizers, which often contribute to soil degradation and nutrient runoff, SeNPs enhance plant metabolism and nutrient absorption while minimizing environmental impact. Their controlled-release properties allow for optimized nutrient delivery, improving crop productivity without the excessive use of synthetic fertilizers. These findings support the application of SeNPs as a sustainable solution in modern agriculture. These results *aestivum* aligning with previous reports (Patil and Chandrasekaran, 2020) that the use of an optimal concentration of nanoparticles can be beneficial for the growth of biological cells and the synthesis of target products, suggesting the versatility of these biogenic nanoparticles and their prospective applications not only in the therapeutic and antimicrobial fields but also in the agricultural and biotechnology sectors, where they could be employed to boost the productivity and yield of economically important microbes and plants. SeNPs promoted *T. aestivum* growth beyond 10 μM, and further increases in SeNP concentration (25–100 μM) resulted in reduced plant growth, likely due to selenium toxicity, oxidative stress, or interference with nutrient uptake, as previously reported in high-dose nanoparticle applications in plants (Zeeshan et al., 2022; Rai et al., 2023). The application of SeNPs at 10 μM significantly increased primary metabolite production in wheat (*Triticum aestivum*), with total soluble sugars reaching 54.32 mg/g and soluble proteins increasing to 139.66 mg/g, promoting both shoot and root growth. Similar study stated that Foliar treatments of Se-NPs on cowpea seeds yielded elevated amounts of Total Carbohydrate and Crude Protein compared to the controls (Samynathan et al., 2023). Elevated protein levels improve the nutritional value of wheat, benefiting both human consumption and animal feed (Khalid et al., 2023). Enhance optimal growth, enzymatic function, and stress resistance. Proteins enhance the plant’s ability to withstand abiotic (drought, salinity) and biotic (pathogens) stressors (Kumari et al., 2022). Elevated sugars, including glucose and fructose, augment the energy content and sweetness of grains, simultaneously supplying energy for growth and development, which results in increased biomass and grain output (Dennis et al., 2019). Sugars serve as osmolytes, aiding plants in coping with stress conditions (Kumari et al., 2022). SeNPs are likely absorbed by plants through root uptake via endocytosis or passive diffusion, followed by transport through the xylem to aerial parts. Inside the plant, SeNPs may undergo biotransformation into selenite (SeO₃^2−^) or selenate (SeO₄^2−^), which can be assimilated into organic selenium compounds such as selenomethionine and selenocysteine, contributing to plant growth and stress tolerance (Hu et al., 2018).

The GC-mass analysis of the *P. ostreatus* extract identified 10 bioactive compounds, with R-Limonene (37.34%) and 9,12-Octadecadienoyl chloride (Z,Z)- (36.80%) being the major components. R-Limonene was documented to possess antioxidant and anti-inflammatory effects (Santana et al., 2020), while 9,12-Octadecadienoyl chloride (Z,Z)- has been associated with antioxidant, anticancer, and thyroid inhibitory activities (Khalid et al., 2022).

Previous studies have identified the existence of diverse metabolites and bioactive compounds within numerous mushrooms and plants (Ferdinand et al., 2020; Venturella et al., 2021; Hamad et al., 2022). These compounds have been linked to diverse biological properties, including antimicrobial, anti-inflammatory, antioxidant, and anticancer activities. The volatile bioactive components discovered in the *P. ostreatus* extract are likely to play significant roles in the investigated biological properties, such as the antioxidant, antimicrobial, and growth-promoting properties of the biosynthesized selenium nanoparticles. The synergistic or individual contributions of these bioactive compounds may contribute to the overall efficacy and multifunctional nature of the myco-synthesized nanoparticles, highlighting the potential of natural sources like *Pleurotus ostreatus* for the development of innovative and sustainable nanomaterials.

The molecular docking studies provide insight into possible mechanisms of action for selenium nanoparticle (SeNP) bioactivity. Favorable binding poses below −3 kcal/mol for targets like AcrB, MmpL3, CYP51, spike protein and photosystem II D1 indicate stable complex formation via hydrogen bonding and hydrophobic contacts, while scores near −3 kcal/mol also imply functional interactions for peroxiredoxin-5 and GA receptor GID1. Predicted interactions with efflux pumps AcrB and MmpL3 could reduce multi-drug resistance by hindering substrate efflux and blocking CYP51’s active site may inhibit fungal growth. Binding to peroxiredoxin-5 enhances the antioxidant potential and interactions with the SARS-CoV-2 spike protein warrant investigation into antiviral effects. The molecular docking study suggests that SeNPs may influence growth processes in algae and plants. The interaction with photosystem II protein D1 and GA receptor GID1 could positively impact Corella growth, while gibberellin signaling could modulate plant growth regulation. Sarkar et al. (2023) explored the biogenic synthesis of selenium nanoparticles (SeNPs) using microorganisms like fungi, bacteria, and plants. They explored SeNPs as potential drug delivery agents through molecular docking studies. SeNPs have antibacterial, antiviral, and antifungal properties due to their interaction with microbial cell membranes and walls. Studies show that biogenic SeNPs reduce plant disease frequency and increase crop yield and quality, making them promising for safer pest and disease control in agriculture compared to chemical treatments.

Biosynthesized selenium nanoparticles (SeNPs) showed promise in antimicrobial applications, plant growth, and environmental remediation, but their long-term effects and optimal use require further study. One key area for future investigation is the cytotoxicity profile of SeNPs, particularly in comparison to conventional selenium sources, with future studies focusing on *in vitro* cytotoxicity assays to determine safe dosage ranges and assess potential toxicity over prolonged exposure. Another critical aspect is the mechanistic validation of SeNPs interactions within biological systems, where experimental validation using binding affinity studies, cellular uptake analysis, and biochemical assays will be necessary to confirm these interactions and better understand their biological effects. Additionally, the uptake, metabolism, and bioaccumulation of SeNPs in wheat plants require further exploration, including how SeNPs are absorbed through root systems, their biochemical transformation within plant tissues, and their potential effects on growth and metabolism using elemental mapping and metabolic profiling techniques. From an ecological perspective, the potential accumulation of SeNPs in ecosystems and their impact on non-target organisms necessitates deeper analysis, with research focusing on bioaccumulation, degradation rates, and ecotoxicological effects through controlled soil and water studies. The mechanism of nanoparticles toxicity may lead to membrane rupture, protein oxidation, and interference with energy transduction, genotoxicity, the release of toxic components, and the generation of reactive oxygen species (ROS) (Morad et al., 2022). Understanding their interactions with beneficial soil microbes and plant-microbe symbiosis will provide valuable insights into their broader ecological implications. Addressing these research gaps will enhance the safe and effective application of SeNPs in medicine, agriculture, and environmental management, ensuring their long-term sustainability and minimal ecological risk.

## 5 Conclusion

This study demonstrated the successful mycosynthesis of selenium nanoparticles (SeNPs) using the aqueous extract of the edible mushroom *P. ostreatus*. The comprehensive characterization of the synthesized SeNPs reveals their spherical morphology, moderate stability, and enhanced antioxidant, antimicrobial, and antiviral properties. Importantly, the SeNPs also exhibited the ability to promote the growth of the crop plant *T. aestivum*, suggesting their potential applications in agriculture. The findings of this study provide to the developing field of knowledge on the utilization of fungal resources for the sustainable production of versatile and eco-friendly selenium-based nanomaterials.

## Data Availability

The original contributions presented in the study are included in the article/supplementary material, further inquiries can be directed to the corresponding author.
